# Elucidating the role of water in collagen self-assembly by isotopically modulating collagen hydration

**DOI:** 10.1073/pnas.2313162121

**Published:** 2024-03-07

**Authors:** Giulia Giubertoni, Liru Feng, Kevin Klein, Guido Giannetti, Luco Rutten, Yeji Choi, Anouk van der Net, Gerard Castro-Linares, Federico Caporaletti, Dimitra Micha, Johannes Hunger, Antoine Deblais, Daniel Bonn, Nico Sommerdijk, Andela Šarić, Ioana M. Ilie, Gijsje H. Koenderink, Sander Woutersen

**Affiliations:** ^a^Van ’t Hoff Institute for Molecular Sciences, Department of Molecular Photonics, University of Amsterdam, Amsterdam 1090 GD, The Netherlands; ^b^Institute of Science and Technology Austria, Division of Mathematical and Physical Sciences, Klosterneuburg 3400, Austria; ^c^University College London, Division of Physics and Astronomy, London WC1E 6BT, United Kingdom; ^d^Electron Microscopy Center, Radboud Technology Center Microscopy, Department of Medical BioSciences, Radboud University Medical Center, Nijmegen 6525 GA, The Netherlands; ^e^Max Planck Institute for Polymer Research, Molecular Spectroscopy Department, Mainz 55128, Germany; ^f^Department of Bionanoscience, Kavli Institute of Nanoscience Delft, Delft University of Technology, Delft 2628 HZ, The Netherlands; ^g^Van der Waals-Zeeman Institute, Institute of Physics, University of Amsterdam, Amsterdam 1090 GL, The Netherlands; ^h^Amsterdam University Medical Centers, Human Genetics Department, Vrije Universiteit, Amsterdam 1007 MB, The Netherlands; ^i^Amsterdam Center for Multiscale Modeling, University of Amsterdam, Amsterdam 1090 GD, The Netherlands

**Keywords:** collagen tissue, hydration, molecular structure, mechanics

## Abstract

Water is known to play an important role in collagen self-assembly, but it is still largely unclear how water–collagen interactions influence the assembly process and determine the fibril network properties. Here, we use the H_2_O/D_2_O isotope effect on the hydrogen-bond strength in water to investigate the role of hydration in collagen self-assembly. We dissolve collagen in H_2_O and D_2_O and compare the growth kinetics and the structure of the collagen assemblies formed in these water isotopomers. Surprisingly, collagen assembly occurs ten times faster in D_2_O than in H_2_O, and collagen in D_2_O self-assembles into much thinner fibrils, that form a more inhomogeneous and softer network, with a fourfold reduction in elastic modulus when compared to H_2_O. Combining spectroscopic measurements with atomistic simulations, we show that collagen in D_2_O is less hydrated than in H_2_O. This partial dehydration lowers the enthalpic penalty for water removal and reorganization at the collagen–water interface, increasing the self-assembly rate and the number of nucleation centers, leading to thinner fibrils and a softer network. Coarse-grained simulations show that the acceleration in the initial nucleation rate can be reproduced by the enhancement of electrostatic interactions. These results show that water acts as a mediator between collagen monomers, by modulating their interactions so as to optimize the assembly process and, thus, the final network properties. We believe that isotopically modulating the hydration of proteins can be a valuable method to investigate the role of water in protein structural dynamics and protein self-assembly.

## Significance

Water influences the self-assembly of collagen, the most ubiquitous protein in our body, but how this happens is still largely unclear. By using a unique, isotope-based method to specifically modulate the water–collagen interaction, we find that water guides the self-assembly process by modulating the attractive interactions between collagen molecules. Our findings suggest that small changes in hydration might be critical in determining tissue dysfunction in collagen diseases, and they suggest a new method to design collagen-based biofunctional materials by isotopically fine-tuning solvent–collagen interactions. This isotopic method of modulating protein hydration can also be used to study the role of water in other self-assembling proteins for which water is involved in the self-assembly process.

Collagen is the main component of connective-tissues such as skin, arteries, and bones, imparting to these tissues the mechanical integrity and properties required to ensure their biological functionality (1). The most abundant collagen type in our body is Type I. The Type I collagen chain contains around 1,000 amino acids and is composed of Glycine(Gly)-Xaa-Yaa repeat units, where Xaa-Yaa are often Proline (Pro) and Hydroxy-proline (Hyp). Three polypeptide strands, adopting a left-handed polyproline II-type (PPII) conformation, further associate to form the typical triple helix, tropocollagen (2). Tropocollagens (collagen monomers) self-assemble to form intermediate fibrillar structures (microfibrils) that further associate into fibrils with an ordered molecular packing structure that maximizes fibril strength (1). The significance of this packing process is clearly shown in connective-tissue diseases in which it is disturbed due to genetic defects in the collagen type I genes; as a result, the mechanical integrity of diverse tissue types is affected (3).

During the self-assembly process, water interacts with collagen, thereby influencing the mechanical properties of the final network. Water surrounds the collagen, and tightly binds to it creating a well-ordered hydration shell (or hydration layer) (4, 5) that controls collagen properties. Water is believed to contribute to the structure and stability of the triple helix via the formation of water bridges (6–10), and it mediates collagen inter- and intra-molecular interactions (11, 12). In particular, the water layer surrounding the collagen monomers has been suggested to create a repulsive interaction (“hydration force”) between collagen molecules, arising from the reorganization of the hydration layer that is required for collagen molecules to approach each other closely (12, 13). Research on collagen hydration has focused mostly on the effect of co-solvents, such as ethanol, propanol or glycerol, on the swelling properties of reconstituted fibril films (14, 15). These solvents, however, differ significantly from water, and having very different molecular sizes and dielectric constants than water, they modify not only the collagen hydration but also many other properties. Despite the evident importance of hydration for the properties of collagen fibrils, the mechanism by which water impacts collagen assembly is still largely unclear.

In this work, we study the role of water–collagen interactions on collagen assembly by replacing water with heavy water (D_2_O). The hydrogen bonds between D_2_O molecules are stronger (by ∼10%) than the ones between H_2_O molecules (16, 17). However, contrary to the solvents used previously to investigate collagen hydration, H_2_O and D_2_O have the same electronic structure, and nearly identical molecular size and dielectric constant (78.06 and 78.37, respectively) (18). Hence, changing the isotopic composition of the water can be used to modulate collagen–water interactions, and so study their effect on the assembly process without affecting the electrostatic interactions due to changes in the solvent dielectric constant. A significant effect of D_2_O on protein self-assembly has been recently observed for α-synuclein (aS) and insulin (INS) (19, 20). In these studies, it was suggested that in D_2_O specific folded structures are stabilized, accelerating (in the case of aS) or slowing down (in the case of INS) the assembly.

Here, we find that the assembly of collagen occurs ten times faster in D_2_O than in H_2_O. This acceleration is somewhat similar to that observed previously for aS (19), but must have a different origin: collagen has a more stable native ordered structure than aS, and (unlike aS) no drastic refolding of the protein is required for initiating the fibrilization (this refolding being a rate-limiting step for the fibrilization of aS). By combining infrared spectroscopy with atomistic simulations, we find that the faster self-assembly observed for collagen in D_2_O is due to the lowering of the energetic penalty of water removal and reorganization at the water–collagen interface, resulting in the enhancement of the initial nucleation rate. Coarse-grained simulations show that the different assembly growth rate and structure in D_2_O can be reproduced by enhancing the electrostatic interactions, which appear to be largely affected by the desolvation energy, and to be a central element driving the initial nucleation. Our results thus suggest that water guides collagen assembly by slowing down the fibril nucleation by moderating the attractive interactions between collagen monomers through the creation of a desolvation energy barrier.

## Results

### Network and Fibril: Kinetics and Structure.

**Fig. 1.**
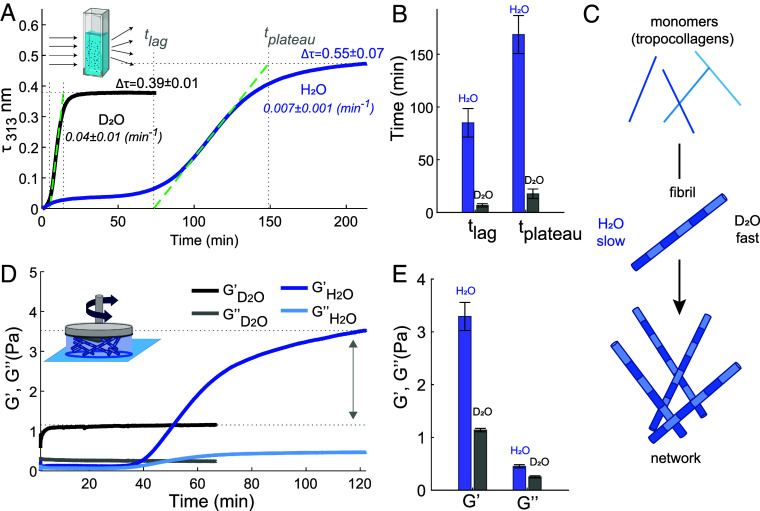
Differences in collagen assembly kinetics and collagen-network elastic properties in H_2_O and D_2_O. (*A*) Turbidity measurements for water and heavy water solutions containing Type I full-length collagen at a concentration of 0.1 mg/ml measured at a temperature of 23 °C. Spectra were collected every 15 s and 30 s for D_2_O and H_2_O experiments, respectively. Final turbidity values and errors represent the mean and standard deviations obtained over 3 different measurements. (*B*) Lag and plateau time values found for collagen fibrilization in H_2_O and D_2_O as described in *SI Appendix*. (*C*) Schematic of collagen assembly in water and heavy water. (*D*) Rheology measurement for water and heavy water solutions containing collagen at a concentration of 0.5 mg/ml. Measurements were conducted at a strain amplitude of 0.8%, an oscillation frequency of 0.5 Hz and temperature of 23 °C. (*E*) Elastic and viscous moduli after attaining the plateau level.

We first study the influence of the isotopic water composition on the kinetics of collagen self-assembly and on the collagen structure at the fibril and network level. We investigate the self-assembly kinetics of collagen in H_2_O and D_2_O by using turbidimetry, a standard method (21–24) that relies on the increase in light scattering as the collagen monomers aggregate into fibrils or fibers (*Inset* of Fig. 1*A*). Fig. 1*A* shows the turbidity-time curves measured in heavy water and water at a collagen concentration of 0.1 mg/ml. Both turbidity profiles show the typical sigmoidal growth profile, characterized by a lag phase of near zero turbidity followed by a growth phase with rapidly increasing turbidity. During the lag time ($t_{\mathrm{lag}}$), collagen aggregates grow primarily in length but little in diameter, forming nuclei which have little ability to scatter light. Subsequently, during the growth phase, the collagen monomers anchor onto collagen nuclei, forming fibrils that quickly grow in diameter and molecular weight at a specific growth rate ($k_{g}$). When the monomers are depleted, the plateau phase is reached ($t_{\mathrm{plateau}}$) as the fibrils attain their mature state (24). The turbidity profiles show that D_2_O samples fibrillate much faster than the H_2_O samples, somewhat similar to α-synuclein in D_2_O and H_2_O (19). The $t_{\mathrm{lag}}$ and $t_{\mathrm{plateau}}$ for collagen assembly are ten-fold shorter in H_2_O than in D_2_O (Fig. 1*B*), and $k_{g}$ is one order of magnitude larger in D_2_O with respect to H_2_O. In addition, the final turbidity value, Δτ, is reduced from 0.55 ± 0.07 to 0.39 ± 0.01 in D_2_O, suggesting that D_2_O favors the formation of thinner collagen fibrils (25) (this will be further investigated below). Similar to the effect of temperature in H_2_O (25, 26), collagen fibrillation in D_2_O accelerates when the temperature is raised from room temperature to 37°C (*SI Appendix*, Fig. S2). Adding salt slows down the assembly in both H_2_O and D_2_O as already reported for H_2_O in previous studies (27–29), but interestingly, with a larger effect in heavy water.

We then performed rheology measurements to monitor the time-dependence of the mechanical response of the collagen solution during self-assembly. The time-dependent elastic and viscous moduli ($G^{\prime }$ and $G^{\prime \prime }$, respectively) of a 0.5 mg/ml collagen solution in H_2_O and D_2_O (Fig. 1*D*) show that collagen gelates faster in D_2_O as compared to H_2_O, as the elastic modulus reaches its plateau value earlier, consistent with the turbidity measurements. Furthermore, the final elastic modulus in water is ∼400% larger in water than in heavy water, (Fig. 1*E*) indicating that the network is much softer in D_2_O. Rheological and turbidity experiments in mixed H_2_O:D_2_O (1:1 volume ratio) indicate that D_2_O-induced changes are D_2_O-concentration dependent, with a significant effect already when ∼50% of H_2_O is replaced by D_2_O (*SI Appendix*, *HDO Measurements* and Figs. S1 and S2). Additional frequency-sweep oscillatory rheology measurements reveal that the dynamics of the network relaxation is not influenced by the presence of D_2_O (*SI Appendix*, Fig. S2*B*).

**Fig. 2.**
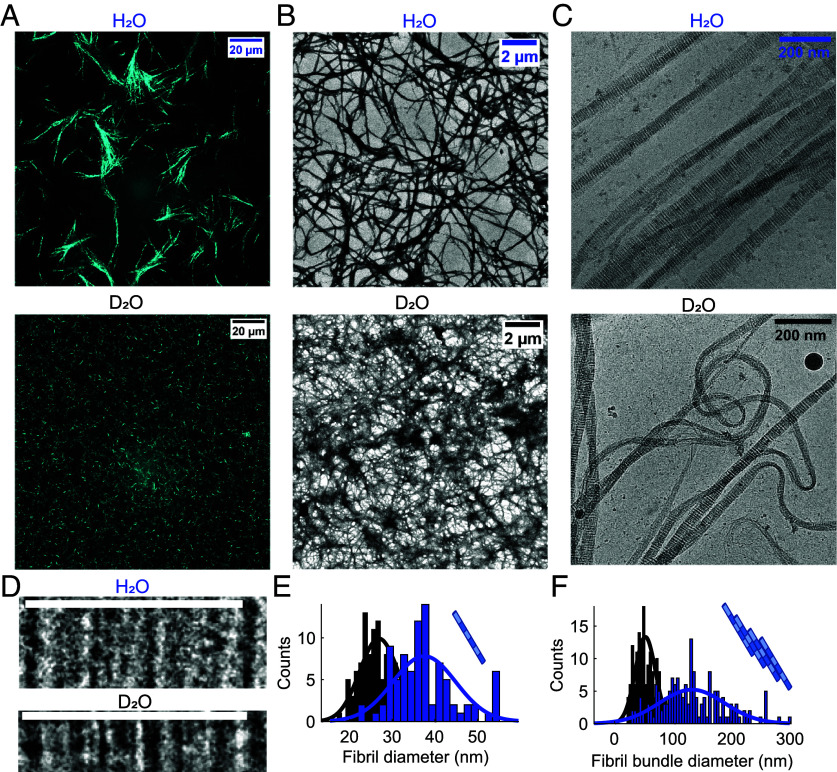
Differences in the collagen fibril and network structures in H_2_O and D_2_O. (*A*) Representative CRM images of the collagen network formed in water and heavy water at a concentration of 1 mg/ml. (*B* and *C*) Representative TEM and cryo-TEM images of the collagen network formed in water and heavy water at a concentration of 1 to 1.25 mg/ml. (*D*) cryo-TEM Images of single fibrils (*Top*, scale bar corresponds to 67.5 nm). (*E*) Distribution of the fibril thickness in H_2_O and D_2_O as calculated from cryo-TEM images. (*F*) Distribution of the fibril bundle thickness (i.e., fibers) in H_2_O and D_2_O as calculated from TEM images.

To investigate the effects of heavy water on the collagen network and fibril, we used confocal microscopy in reflectance mode (CRM) to obtain images of the networks (Fig. 2*A*). In water, collagen networks are isotropic and exhibit fan-shaped bundles of fibrils and large pore spaces, similar to the micro-structures observed for rat tail Type I collagen in previous studies (25, 30). By contrast, gelation in heavy water does not lead to bundling observable at the micrometer scale, and instead a uniform and dense distribution of thin fibers is observed. To resolve the structure of the single fibrils, and to investigate whether D_2_O affects the staggered arrangement of collagen, we performed cryo-TEM experiments (Fig. 2 *C* and *D*). Fig. 2*C* shows images of uranyl-stained fibrils (taken from cryo-TEM images) assembled in H_2_O and D_2_O. Fig. 2*D* shows zoomed-in images of single fibrils. We observe the characteristic patterns of thin, stained sub-bands caused by the alignment of charged sidechains in the fibril (31). These sub-bands are expected to repeat at a periodicity (the D-band periodicity) of about 67 nm (32). The D-banding is characteristic of collagen fibrils, and it arises because collagen molecules follow a specific quarter-staggered fashion packing. To establish whether heavy water affects D-band periodicity, we measured it for fibrils assembled in the two water isotopomers (*SI Appendix*, Fig. S5). We find the same values in D_2_O and H_2_O (67.3 ± 1.3 nm and 67.0 ± 0.8 nm, respectively), indicating that heavy water does not affect the quarter-staggered assembly of collagen. In addition, cryo-TEM reveals that collagen self-assembles into thinner fibrils in D_2_O than in H_2_O: the diameter distributions are centered at 27 nm (σ = 4 nm) and 38 nm (σ = 7 nm) for collagen fibrils assembled in D_2_O and in H_2_O, respectively (Fig. 2*E*). Similarly, quantitative analysis of the TEM images shows that the fibers (i.e., fibril bundles) formed in heavy water are much thinner (Fig. 2*F*): the average bundle diameter is 132 nm (σ = 55 nm) in H_2_O [in agreement with ref. 25] and 52 nm (σ = 18 nm) in D_2_O. In summary, imaging techniques show that collagen assembles in the quarter-staggered arrangement in both H_2_O and in D_2_O, but in D_2_O it forms thinner fibrils and fibers (in agreement with the turbidity measurements), resulting in a more uniform network at the micrometer scale.

### Water–Collagen Interactions.

**Fig. 3.**
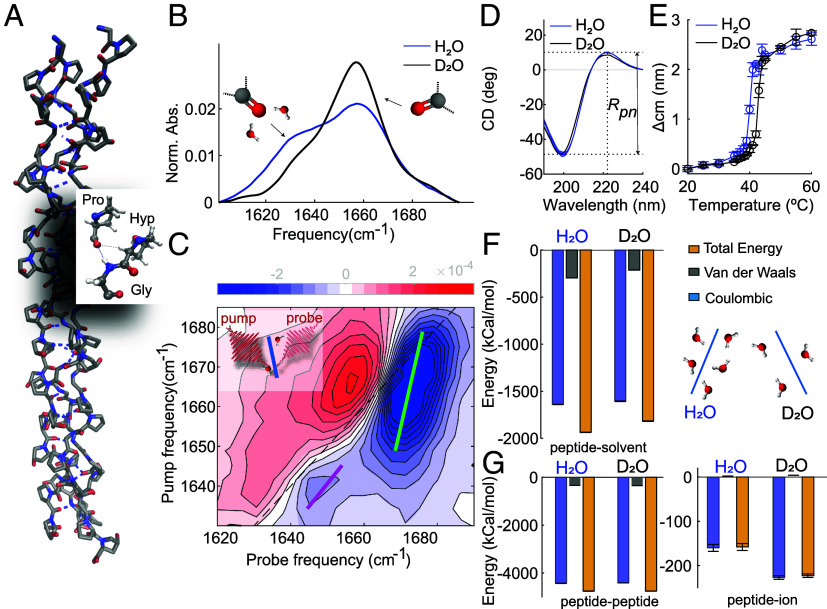
Collagen is less hydrated in D_2_O than in H_2_O, but retains the same helicity. (*A*) Crystal structure of the (Gly-Pro-Hyp) nonamer PDB ID: 3B0S(33). (*B*) IR spectra of heavy water and water solutions containing Type I full-length collagen at a concentration of 2 mg/ml and 10 mg/ml, respectively, recorded at 23°C. Full IR spectra are shown in *SI Appendix*, Fig. S3. The IR spectrum in D_2_O was obtained using FTIR in transmission mode, in H_2_O it was obtained by using FTIR in reflection mode (ATR-FTIR). In the latter case, because of the shorter optical path length, we used a higher collagen concentration to obtain a sufficient signal-to-noise ratio. The IR spectrum of collagen in water is not concentration dependent (ref. 34 and *SI Appendix*, Fig. S4). (*C*) 2D–IR spectrum of a heavy water solution containing Type I full-length collagen at a concentration of 2 mg/ml recorded at a waiting time between pump and probe pulses of 1 ps. The blue contours represent a decrease in absorption (ΔA < 0) due to depletion of the v = 0 state, and the red contours an increase in absorption (ΔA > 0) due to the induced absorption of the v = 1 → 2 transition. Colored lines in the 2D-IR spectrum represent the calculated central lines (See *SI Appendix* for more details). (*D* and *E*). CD spectra and melting curves extracted from temperature-dependent CD measurements of Type I full-length collagen dissolved in water and heavy water at a concentration of 0.1 mg/ml, respectively (see *SI Appendix* for more details). (*F*) Interaction energies between the peptide and the solvent (D_2_O, H_2_O) molecules and schematic of collagen hydration in D_2_O and H_2_O. (*G*) Intramolecular energies and energies between the peptide and the ions in D_2_O and H_2_O.

To understand the molecular origin of the differences in collagen-assembly kinetics and structure in D_2_O and H_2_O, we compare the structure and hydration of monomeric collagen (its triple-helix structure is shown schematically in Fig. 3*A*) in H_2_O and D_2_O using infrared (IR), two-dimensional IR (2D-IR), circular dichroism (CD) spectroscopy and molecular dynamics (MD) simulations. IR and 2D-IR spectroscopy probe the local structure and solvation by studying the infrared absorption bands of amide I modes (33–35), CD spectroscopy probes the helicity (36, 37) and the stability (38) of collagen, and MD simulations provide insight into the structural details of the monomer in different solvents.

In Fig. 3*B*, we report the normalized IR spectra of the triple-helical collagen monomer dissolved in D_2_O and H_2_O recorded at a temperature of 23 ^°^C. We observe two main bands at 1,635cm^−1^ and 1,660 cm^−1^, in agreement with literature (39–42) with the 1,660 cm^−1^ band more intense in heavy water with respect to water. Since carbonyl groups in the collagen chain experience different amounts of solvent exposures (10), the low frequency band was previously assigned to the vibrations of carbonyl that are accessible and well-exposed to water (42). To verify this assignment we use 2D-IR spectroscopy, a technique that can provide direct information on the collagen hydration (34). In pump–probe 2D-IR spectroscopy, we use a tunable narrow-band pump pulse to excite molecular vibrations at a specific frequency $\nu_{\mathrm{pump}}$, and measure the pump-induced change in absorption ΔA at all frequencies using a broad-band probing pulse. Each vibrational mode of a molecule gives rise to a +/− doublet on the diagonal (34). In the 2D-IR spectrum reported in Fig. 3*C*, we observe two pairs of diagonal peaks at pump frequencies of 1,635cm^−1^ and 1,660 cm^−1^. The lineshapes of the two diagonal peaks differ significantly, with the 1,660 cm^−1^ diagonal peak being more tilted with respect to the diagonal. The dependence of the 2D-IR response on the pump frequency is a measure of the inhomogeneous broadening of the IR band (34), which is due to a distribution of transition frequencies caused by solvent–protein interactions. The degree of inhomogeneity can be characterized by calculating the inverse value of the slope of the 2D-IR bleaches (central line slope or CLS) (43); and we find that the CLS values for the 1,635 and for the 1,660 cm^−1^ peaks are 0.7 ± 0.15 and 0.35 ± 0.11, respectively (values and errors represent the mean and standard deviations obtained over 3 different measurements). The higher CLS value for the band at low frequency indicates a larger inhomogeneity, and thus a broader frequency distribution, than for the peak at 1,660cm^−1^. The broader frequency distribution is due to interactions between functional groups and solvent molecules, indicating that the amide groups absorbing at 1,635cm^−1^ experience better solvation than the ones absorbing at 1,660 cm^−1^. We then fit the IR spectra in D_2_O and H_2_O (Fig. 3*B*) by using Gaussian-shaped peaks (see *SI Appendix*, Fig. S4 *C* and *D* for more details). We found that the area of the peak of the more solvent-exposed carbonyl decreases by ∼30% in intensity when collagen is dissolved in D_2_O as compared to H_2_O. This spectral difference [also observed in ref. 41) was found to be independent of collagen concentration and amide H/D exchange (*SI Appendix*, Fig. S4 *D*–*F*). Furthermore, an increase in the ratio between less- and well-solvated carbonyl bands is observed in collagen fibril solutions when the fibrillation takes place in D_2_O (*SI Appendix*, Fig. S4*A*); but also in collagen dissolved in H_2_O (with the temperature set to 4 ^°^C to prevent fibrillation) (40).

We investigated whether the reduced hydration in D_2_O influences the helicity of the collagen triple helix using CD spectroscopy. Fig. 3*D* shows the CD spectra of collagen dissolved in water and heavy water at a concentration of 0.1 mg/ml. Both CD spectra have a minimum at 198 nm and a maximum at 220 nm, the typical spectral signatures of the collagen triple-helix (36). To check whether the reduced solvation affects the collagen helicity, we calculated the ratio between the intensities of the maximum and the absolute of the minimum values, $R_{\mathrm{pn}}$ (an experimental criterion for triple-helicity) (37). We found identical ratios (∼0.19) in H_2_O and D_2_O, indicating a similar helicity of collagen. In addition, we extracted the melting temperatures of the collagen triple helix from the temperature dependence of the CD spectra (see Fig. 3*E* and *SI Appendix* for more details), resulting in 40 ± 1 °C and 43 ± 1 °C in H_2_O and D_2_O, respectively. This result indicates that the collagen monomer has a less stable structure in H_2_O than in D_2_O, in agreement with previous studies on collagen Type I and collagen-based peptides (38).

To explain the microscopic origin of the experimentally observed reduction in water–collagen interactions in D_2_O compared to H_2_O, we performed molecular dynamics simulations of the (Gly-Pro-Hyp) nonamer triple helix starting from the crystal structure PDB ID: 3B0S (44), Fig. 3*A*. Two sets of simulations were carried out in H_2_O and D_2_O at 300 K. In each case, five independent copies were run for a cumulated sampling time of 10 μs. The triple helices are structurally stable over the course of the simulations with an average RMS deviation from the crystal structure of 2.4 ± 0.02 Å. The energetic analysis reveals that the total interaction energies between the nonamer and the solvent are less favorable in D_2_O than in H_2_O (−1,818±3 kcal/mol in D_2_O and −1,936±2 kcal/mol in H_2_O, Fig. 3*G*). The reduction of water–protein interaction is also reflected in the smaller number of solvent–collagen hydrogen bonds in deuterated water (131 ± 0.4) compared to water (136 ± 0.4). The reduced solvation in D_2_O is also subtly mirrored in the radial distribution functions (RDF) of water around Hyp, Gly, and Pro (*SI Appendix*, Fig. S9), and even more so in the RDF of water around the carbonyl groups, which shows a reduction in the first hydration shell when water is replaced with heavy water (*SI Appendix*, Fig. S10), confirming the reduced hydration in D_2_O that is experimentally observed using IR spectroscopy. The intramolecular energies (Fig. 3*F*) and number of intramolecular hydrogen bonds are essentially identical, while the interaction with the ions is more favorable in D_2_O (−223±4 kcal/mol) than in H_2_O (−158±8 kcal/mol). The latter finding is ascribed to the reduced hydration in D_2_O as compared to H_2_O and comes as a consequence of a tighter network of H-bonds (94,005 ± 4 and 92,940 ± 2, respectively). Thus, the molecular dynamics simulations show a reduction in water–collagen interactions in D_2_O as compared to H_2_O in agreement with previous results on other biomolecules (45, 46), leading to a less solvent-exposed and more stable protein structure without significantly altering the collagen helicity.

### Collagen-Collagen Interactions.

**Fig. 4.**
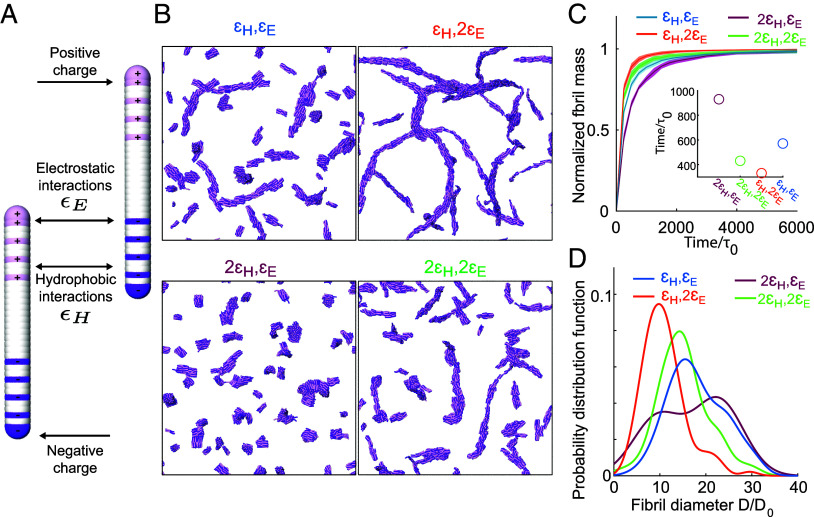
Coarse-grained simulations can qualitatively reproduce the differences in collagen fibril and network structure in H_2_O and D_2_O. (*A*) The collagen-mimetic molecules are simulated as elastic rods made of overlapping beads. The beads carry charges as indicated (positive are pink, negative are purple, and white is neutral). On top of these electrostatic interactions, all the beads between different molecules interact via generic hydrophobic interactions. (*B*) Simulation snapshots of the equilibrated system for different combinations of electrostatic and hydrophobic interactions (ϵH = 0.05kT, ϵE = 5kT). (*C*) Normalized fibril mass as a function of simulation time. The *Inset* shows the time at which the assembled mass reaches 80% of the total monomer mass, where $\tau_{0}$ is the MD unit of time. (*D*) Probability distribution function of the fibril diameter D normalized by the smallest measured fibril diameter $D_{0}$.

How can partial dehydration of collagen in D_2_O modify the collagen-collagen interactions in such a way as to cause the observed changes in assembly kinetics and structure? To address this question, we performed coarse-grained molecular dynamics simulations using collagen-mimetic molecules ( 47). The assembly of collagen is known to be driven and regulated by an interplay between hydrophobic and electrostatic interactions (14, 27, 48–54), and coarse-grained simulations have proven successful in revealing how this interplay controls the self-assembly (55). To see which of these forces is most strongly influenced by the reduced hydration in D_2_O, we systematically modify them in the simulations and see whether we can reproduce the experimentally observed changes in collagen assembly rate and fibril structure. In our coarse-grained MD simulations, collagen molecules are described as elastic rods that carry a pattern of charges (Fig. 4*A*). The rods can interact with each other via screened electrostatic interactions as well as via generic, hydrophobic-like attractions and have previously been shown to form clusters and collagen-like fibrils (55). Electrostatic interactions are modeled using a Debye–Hückel potential, while a Lennard-Jones potential is used for hydrophobic interactions. To study how the reduced hydration in D_2_O can cause the observed changes in assembly rate and fibril structure, we vary the strength of electrostatic and hydrophobic interactions, and average the obtained results over ten independent simulation runs for each set of parameters. Snapshots of these simulations are shown in Fig. 4*B*. The results (Fig. 4*C* and *D*) show that increasing the hydrophobic interaction strength decreases the assembly rate and increases fibril diameter, the opposite of the experimentally observed trend. However, upon increasing the electrostatic interaction strength, the assembly rate is increased and fibril diameter is decreased, exactly as is observed experimentally in D_2_O (Figs. 1 and 2). These results indicate that the experimentally observed acceleration of assembly as well as the thinner fibrils in D_2_O compared to H_2_O can be effectively reproduced by enhancing electrostatic interactions, rather than by enhancing hydrophobic interactions.

## Discussion

Our results show that changing the solvent from H_2_O to D_2_O causes a ten-fold acceleration of the collagen assembly (Fig. 1), and a dramatic structural change and softening of the final fibril network (Fig. 2). These differences become more pronounced with increasing D_2_O concentration, with a significant effect on the kinetics and mechanical properties already at a 1:1 ratio of H_2_O:D_2_O (*SI Appendix*, Figs. S1 and S2). In the nucleation-growth mechanism of collagen self-assembly, the fibril diameter is determined mainly during the nucleation step (56). Our coarse-grained simulations suggest that in D_2_O more nucleation centers form due to the enhancement of electrostatic interactions between collagen monomers (Fig. 4). These nucleation centers compete with each other for the remaining collagen monomers, so that the increased attractive interaction in D_2_O results in the thinner fibrils (and fibers) that are observed in the cryo-TEM images and the coarse-grained simulations. This is consistent with the thinner collagen fibrils formed at higher temperature (25), which also accelerates fibrilization (30). By combining (2D-)IR with CD measurements and MD simulations we find that water–collagen interactions are reduced in D_2_O, leading to a more stable and less water-bound structure, without altering the collagen helicity. These findings are consistent with previous studies which have shown that D_2_O is a poorer protein solvent than H_2_O, and that D_2_O favors a less water-exposed but more stable protein structure (38, 57–66), affecting the assembly properties (19, 20, 67) (see ref. 68 for more details).

How can a reduction in collagen hydration affect the assembly process and the fibril structure so dramatically? For proteins to interact, the water molecules, which are tightly bound to the hydrophilic groups on the protein surface, have to be released from the protein surface and reorganized, leading to a large energetic penalty (desolvation energy) for the protein assembly. It has been suggested that this desolvation energy plays a crucial role in the assembly of amyloid proteins (69, 70) affecting the first stages of the fibril formation: although the assembly into fibrils is thermodynamically favored by the entropic gain in solvent release, the fibril nucleation is limited by the large desolvation energy. The initial self-assembly rate can thus be increased in less hydrating conditions, resulting in a faster assembly.

Our results indicate that the reduced hydration in D_2_O affects the assembly process (and final fibril and network properties) of collagen in a similar manner, by lowering the desolvation energy barrier, which limits the initial nucleation. The coarse-grained simulations show that the acceleration in the initial nucleation rate can be reproduced by the enhancement of the electrostatic interactions, suggesting that these play an important role in determining the speed of the initial nucleation. This scenario would be consistent with previous observations, such as the acceleration of collagen fibrillization observed for collagen Type II (which possesses a larger number of ionizable groups than Type I) (71) and the strong deceleration of collagen assembly when adding monovalent salt, which screens the electrostatic interactions (27–29). In our case, increasing the salt concentration also slows down the collagen assembly, but interestingly, the impact of salt on the lag and plateau times is larger in D_2_O than in H_2_O (*SI Appendix*, Fig. S3). This difference can be attributed to the larger contribution of electrostatic interactions during collagen assembly in D_2_O than in H_2_O. These findings, together with the previously reported role of hydrating water molecules in modulating the electrostatic interactions in proteins (69, 70, 72), support the scenario suggested by the coarse-grained simulations that assembly in heavy water occurs more rapidly due to enhanced electrostatic interactions because of the lower energy barrier in water removal from the collagen surface. A similar explanation has been proposed previously to explain the impact of elevated temperature on collagen assembly (73), which, similarly to replacing H_2_O by D_2_O, leads to faster collagen assembly, thinner fibrils and a softening of the network (25, 26). Although our measurements and prior studies support our hypothesis, we advocate for further studies to specifically investigate the impact of desolvation on the strength of collagen-collagen electrostatic interactions. Additionally, based on our results it is difficult to determine which specific stage of the fibrilization process is mostly affected by the change in electrostatic interaction, and we hope that our results will also inspire further research to clarify this issue.

In earlier studies, it was already suggested that a short-range repulsive “hydration force” might be crucial for the structure and properties of collagen fibrils, and that the penalty associated to restructuring the tightly bound water molecules might prevent collagen molecules from coming too close to each other (12, 13, 15). However, so far this potential role of desolvation energy in collagen assembly was only indicated indirectly in experiments and simulations (14, 49, 50). The unique possibility of isotopically modulating the hydration while keeping the other solvent properties the same makes it possible to directly demonstrate the crucial role of the desolvation in collagen assembly. Our results indicate that water controls the mechanics of collagen networks by moderating attractive interactions between collagen monomers that guide the self-assembly. In this way, water drives the formation of few initial nuclei rather than many competing ones, ensuring the cooperative nature of collagen self-assembly. In the future, it would be interesting to determine whether specific regions in the collagen sequence, and if so which ones, are most important in establishing this mediating role of water. Furthermore, by exploiting the different hydration in D_2_O and H_2_O, we intend to probe this mediating role for collagen interactions with other tissue components, such as minerals in bones (74) and hyaluronic acid in cartilage (75).

Our findings provide insights into how hydration modulates collagen properties to finely tune the mechanics of living tissues (76) and suggest avenues toward the design of artificial collagen-based materials and development of novel drug discovery strategies. Controllable and tunable macroscopic properties might be achieved by subtle changes in the solvent isotopic composition instead of altering the chemical structure of a biomaterial’s building blocks. Furthermore, minimal changes of the solvent conditions can induce the structural rearrangement of a protein (without disrupting its secondary structure) to unveil novel allosteric pockets, hence rendering the target as druggable (77). Finally, altered water–collagen interactions are believed to play a role in several age-related diseases (8, 78–80), and to partially contribute to tissue dysfunction in these disorders. It is well-known that genetic defects in the collagen type I genes COL1A1 and COL1A2 can cause osteogenesis imperfecta, Caffey disease and Ehlers–Danlos syndrome with a distinct bone or skin pathology, but our limited knowledge of the collagen folding hierarchy and its tissue-specific interfering factors makes it difficult to understand the mechanisms leading to such hyperostosis or fragility of bones, skin or blood vessels (81–83). The results presented here show that collagen hydration modulates the assembly rate and diameter of fibrils, properties that are also impacted in these diseases (84–86). It is therefore not unlikely that modified hydration may exacerbate the molecular defects of collagen Type I (i.e., excessive posttranslational modification, misfolding) in determining the phenotypic outcome. We hope that further studies will give insight in the way that water distribution influences collagen quality, and how this might potentially be used for therapeutic purposes (87).

## Conclusion

We have shown that changing the solvent from H_2_O to D_2_O induces a tenfold acceleration of collagen assembly, and leads to thinner fibrils and a much softer collagen network, with significant effects already observable when 50% of H_2_O is replaced by D_2_O. By combining spectroscopy with molecular dynamics simulations, we have found that collagen in D_2_O is less hydrated than in H_2_O, and that it adopts a less water-exposed and more stable structure without altering its helicity. Our results indicate that the kinetic and structural changes originate from a lower energetic penalty for water removal and water reorganization at the collagen surface in D_2_O, and coarse-grained simulations suggest indirectly that this desolvation energy influences mostly electrostatic interactions, which seem to be crucial in determining the nucleation rate. Our results directly demonstrate the role of hydration in collagen self-assembly: The water layer surrounding the collagen acts as a mediator, moderating collagen-collagen interactions in order to slow down the assembly so as to optimize the final network properties.

## Materials and Methods

### Sample Preparation.

Lyophilized collagen containing telopeptides (Type I collagen from rat tail tendon, Roche cat. no. 11179179001, batch numbers: 66914600 and 62193600) was purchased from Sigma Aldrich. Glacial acetic acid was purchased by Sigma (Emsure, no. 1000632500) and sodium hydroxyde (NaOH) was purchased by Honeywell Fluka, no. 319511-500ML. Deuterated materials used are D_2_O (deuterium oxide; Sigma-Aldrich, no. 151882-25G); Acetic acid-d_4_ (Sigma-Aldrich, no. 233315-5G); NaOD (sodium deuteroxide; Sigma-Aldrich, no. 372072-10G). Collagen was dissolved in water, heavy water or 1:1 water:heavy water solutions containing 0.1 or 0.2% (v/v) of acetic acid (acetic acid solutions: pH ∼3.1 to 3.4). The collagen was dissolved in acetic acid solutions to obtain a stock solution of collagen at a concentration of 2 or 2.5 mg/ml. After dissolution, collagen was left to dissolve at room temperature for 1 to 2 h while gently stirring every 10 to 15 min, and then stored at 4 to 6 ^°^C for at least 1 to 2 d before usage ensuring collagen is fully dissolved. The collagen stock solution was further diluted in acetic acid solutions (same that was used for the initial dissolution) to obtain concentrations of 0.2 mg/ml (for turbidity/CD measurements) and 1 mg/ml (for rheology measurements), and left to equilibrate for at least 1 d at 4 to 6 ^°^C before to perform turbidity/rheology measurements. Stock solution of collagen at a concentration of 10 mg/ml in water solution containing acetic acid at a concentration of 0.1 wt% was prepared to perform FTIR-ATR measurements in water. All collagen samples were prepared on ice to prevent early self-assembly and the self-assembly was initiated by neutralizing acidic collagen solutions. First, weighing collagen in an Eppendorf tube and subsequently adding an equal volume of customized buffer solution to obtained a final pH of 7.2 to 7.6 and ionic strength I = 0.17 M. To assure mixing, neutralized collagen solution was quickly pipetted up and down for 10 to 15 times. The customized buffer solution is made of Milli-Q water (or D_2_O), 10× PBS solution, made with phosphate buffered saline tablet (purchased by VWR, no. E404-100TABS) dissolved in 10 ml of Milli-Q or (D_2_O), and 0.1 M NaOH (or NaOD). The customized buffer solution contained a volume fraction of 20% of 10xPBS, whereas the volume ratio Milli-Q:NaOH (or D_2_O :NaOD) was adjusted to obtain collagen solutions with a final pH of 7.2 to 7.6. The Milli-Q:NaOH (or D_2_O:NaOD) volume ratio depends on final collagen concentration, and variation can be observed because of possible differences in the initial concentration of the stock solutions of acetic acid and NaOH (or NaOD). Generally, for collagen dissolved in 0.2% of acetic acid, the Milli-Q:NaOH (or D_2_O :NaOD) volume ratio is around 32 to 40%:48 to 40%, whereas for collagen dissolved in 0.1% of acetic acid, the volume ratio is around 15 to 20%:65 to 60%. For the measurements at different salt concentration, we first determined the required volume ratio for the case of 0 mM of added salt to obtain collagen solutions with a final pH of ∼7.5 (32%:48% for D_2_O samples and 38%:42% for H_2_O samples). Then, stock solutions of NaCl (purchased in solid form from Sigma Aldrich) were prepared in heavy water and water with a concentration of 0.625 ± 5 mM and 0.716 ± 5 mM, respectively, and mixed with the NaOH (or NaOD) and 10XPBS stock solutions used to determine the ratio instead of D_2_O and Milli-Q to obtain a final salt concentration of 150 mM in the neutralized collagen solutions. NaCl stock solutions were then further twofold diluted and mixed with the NaOH (or NaOD) and 10XPBS stock solutions used to determine the ratio instead of D_2_O and Milli-Q to obtain a final salt concentration of 75 mM. The pH of all collagen solutions at different salt and collagen concentrations was measured by using a pH-meter (Thermo Scientific, Orion 2 Star) that was calibrated for measuring the pH in H_2_O solutions instead of D_2_O solutions. The measured pH^∗^ of a D_2_O solution was transformed to the pH value by using the following equation: $pH=pH^{*}\cdot 0.929+0.4\;$ (88).

### Infrared Spectroscopy.

For IR-measurements of heavy water solutions, samples containing collagen at different concentrations (2.5, 2, 1.25 or 0.5 mg/ml) were placed in a circular sample cell composed by two CaF_2_ windows separated by a 100-μm spacer. Measurements were done in transmission mode using a Bruker Vertex 70. Per measurement, 32 scans were made, with a spectral resolution of 2 cm^−1^. The temperature was kept at 23 ^°^C using a temperature controller (Julabo, TopTech F32-ME). The frequency range was from 7,000 cm^−1^ to 400 cm^−1^. For IR-measurements of water solutions, sample containing collagen at a concentration of 10, 5, and 2 mg/ml was measured in reflection mode using a PerkinElmer Frontier FT-IR spectrometer fitted with a Pike GladiATR module equipped with a diamond ATR-crystal (ϕ = 3 mm). Spectra were averaged over 20 scans. Temperature was maintained at room temperature (21 °C) by using a built-in heating/cooling plate. The spectrum of the solvent was subtracted to obtain the individual spectrum of the collagen.

### Two-Dimensional Infrared Spectroscopy.

A detailed description of the setup used to measure the 2DIR spectra can be found in ref. 89. Briefly, pulses of wavelength 800 nm and with a 40 fs duration are generated by using a Ti:sapphire oscillator, and further amplified by using a Ti:sapphire regenerative amplifier to obtain 800 nm pulses at 1 kHz repetition rate. These pulses are converted in an optical parametric amplifier to obtain mid-IR pulses (∼20 μJ, ∼6,100 nm) that has a spectral full width at half max (FWHM) of 150 cm^−1^. The beam is split into a probe and reference beam (each 5%), and a pump beam (90%) that is aligned through a Fabry-Pérot interferometer. The pump and probe beams are overlapped in the sample in an ∼250-μm focus. The transmitted spectra of the probe (T) and reference ($T_{0}$) beams with pump on and off are recorded after dispersion by an Oriel MS260i spectrograph (Newport, Irvine, CA) onto a 2 × 32-pixel mercury cadmium telluride (MCT) array. The probe spectrum is normalized to the reference spectrum to correct for pulse-to-pulse energy fluctuations. The 2DIR signal is obtained by subtracting the probe absorptions in the presence and absence of the pump pulse.

### Circular Dichroism.

CD spectra were recorded with a JASCO CD spectrometer (Model: J-1500-150) in the far-UV at wavelengths, λ, ranging from 180 to 260 nm to obtain information on the secondary structure of the proteins. Data were recorded with a data pitch of 0.2 nm, a scan speed of 20 nm/min, a digital integration time of 0.5 s, and an optical path length of 1 mm. Spectra were smoothened using the Savitzky–Golay filter built-in in the spectrometer software. Temperature-dependent measurements were performed at temperatures ranging from 20 to 60 °C at increments of 5 °C with an equilibration time of 4 min. At 35 to 45 °C smaller increments of 1 °C were used with 8 min equilibration time. From each experiment, the spectrum of the buffer was subtracted and the results of the three experiments were averaged for the final analysis.

### Turbidity.

The kinetics study of collagen self-assembly was performed on a UV-Vis spectrophotometer (Agilent Technologies, Cary 8453). Both collagen and buffer solutions at the desired concentration were placed in the fridge at 4 to 6 ^°^C for 20 min prior starting the turbidity measurement, and transported in a container cooled with ice to the UV/Vis spectrometer lab, where they were neutralized on ice to prevent early self-assembly and the self-assembly was initiated by neutralizing acidic collagen solutions. To insure mixing, neutralized collagen solution was quickly pipetted up and down for 10 to 15 times, and then neutralized cold collagen solutions were pipetted into plastic cuvettes (Brand, UV-Cuvette micro, no. 759220), which were quickly sealed with a cover to avoid evaporation and H/D isotopic exchange and subsequently placed in the water-jacked cuvette holder. Measurements were performed at room temperature (21 °C). Spectra were recorded every 15 s and 60/120 s after neutralization for heavy water and water samples, respectively. The spectrum of the respective solvent was used as a background. As collagen self-assembly proceeded, the absorbance at a wavelength of 313 nm ($A_{313}$) was recorded as a function of time. Increase of $A_{313}$ over time during collagen self-assembly represents an increase in scattering. The absorbance readings were converted into turbidity values (τ) by using the relation: $\tau =A_{313}\cdot ln10$, subtracting the turbidity value at early time before fibrilization started.

### Rheology.

Rheology study of collagen was performed with a stress-controlled rheometer (Anton Paar, Physica MCR 302), equipped with a cone-plate geometry (50 mm diameter, 1^°^ cone angle, 100 μm gap). The bottom plate temperature was controlled using a Peltier element. Neutralized cold collagen solutions at a concentration of 1.25 mg/ml (experiments shown in *SI Appendix*) or 0.5 mg/ml (experiments shown in the main text) were pipetted onto the plate, and the cone was immediately lowered to the measuring position. We used a thin layer of low-viscosity mineral oil (Sigma-Aldrich, no. 330760-1L) around the sample to prevent solvent evaporation and H/D isotopic exchange. Within ∼2 min the oscillatory rheology measurement was started.

### CRM.

To prepare collagen samples for CRM measurements, we used the protocol described in ref. 75. Briefly, neutralized cold collagen solution was pipetted into the customized sample holder, composed of two coverslips and the adhesive silicone isolator (Thermo Fisher Scientific, Press-to-Seal silicone isolator) in between. The coverslips were cleaned beforehand with isopropanol and Milli-Q water and dried by nitrogen flushing. The sample holder was then immediately placed into a petri dish and sealed by parafilm to prevent solvent evaporation and H/D isotopic exchange. Collagen at a concentration of 1 mg/ ml was left to polymerize at 23 ^°^C. Both water and heavy water samples were measured after at least 150 min at 23 ^°^C from neutralization to attain full network formation. The equilibrium collagen network images were taken by an inverted confocal laser scanning microscope (Leica Stellaris 8 platform) equipped with a 63x, NA = 1.30 glycerol-immersion objective (Leica), a (supercontinuum) white light laser with laser line 488 nm for illumination and the reflected light was detected with silicon multi-pixel photon counter (Leica, Power HyD-S) detector. Glycerol (Leica, ISO 836) was used for objective immersion.

### TEM.

To prepare collagen samples for TEM measurements, we used the protocol described in ref. 90. Briefly, after neutralization, fibril assembly was initiated by placing the samples in a closed container (comprised of the cap of a closed Eppendorf tube placed upside down) for at least 150 min. The collagen fibrils were transferred to glow-discharged electron microscopy grid by peeling off the collagen gel drop surface with the grid (purchased from QuantiFoil, C support Cu400), which was left on the collagen surface between 1 and 12 h. The sample was then washed 1 or 2 times by placing a drop of milliQ water and blotting the drop without completely drying the grid. Finally, the sample was stained by adding a drop of 2% uranyl acetate and blotting it to dryness. TEM images were analyzed by using ImageJ, which is an image analysis and open source software (91). After scale calibration, thickness of the fibrils was calculated by taking the width of different fibrils in at least four different images of four different grids for a total of around 150 measured thickness points. Width measurements were taken from the nonsmoothed image by manually drawing a line perpendicular to the long axis of the bundle or the filament between the edges of the fibril. The edges were determined as the location where the darkened region produced by the defocus halo starts.

### cryo-TEM.

Briefly, after neutralization, fibril assembly was initiated by placing the samples in a closed Eppendorf tube letting them assemble for 2.5 to 12 h at room temperature (21 ^°^C). The collagen fibrils were transferred onto glow-discharged 200 mesh gold 2/2 Quantifoil + 2 nm C (purchased from Electron Microscopy Sciences) and incubated for 10 min inside a H_2_O or D_2_O humidity chamber. After incubation the excess liquid was blotted away, the fibrils stained for 2 min with 2% uranyl acetate, washed 3 times, and vitrified using a Vitrobot Mark IV (Thermo Fisher). The cryo-TEM images were acquired using a TALOS F200C-G2 operated at 200 kV and equipped with a Falcon 4i direct electron detector. The cryo-TEM images were analyzed by using ImageJ (91). After scale calibration, fibril diameter was calculated by taking the width of different fibrils in different images of four different samples with a total of 111 and 87 measured thickness points for D_2_O and H_2_O, respectively. Width measurements were then taken from the nonsmoothed image by manually drawing a line perpendicular to the long axis of the filament between the edges of the fibril. One edge was determined at the location where the darkened line produced by the densely packing stained collagen starts and the other edge was determined at the location where a light halo started, indicating an empty region between fibrils, or at the location at which the darkened line changed its orientation, indicating the presence of a second fibril. To determine the D sub-banding pattern, the fibril images were rotated to obtain a horizontally aligned fibril, and, then, an area containing 5 D-repeats (∼300 nm) was selected. By using ImageJ, the intensity profile was then obtained.

### Molecular Dynamics Simulations.

The crystal structure of collagen-mimetic peptides composed of showing nine Gly-Pro-Hyp repeats PDB ID: 3B0S (44) was used as starting conformation. Two sets of simulations of the collagen triple helix {Gly-Pro-Hyp}_9_ were carried out in water and heavy water at 300 K, cumulating 10 μs (5 × 2 μs runs). All simulations were run using the GROMACS 2020.4 software package (92, 93), the CHARMM36m forcefield (94) and explicit solvent molecules, i.e., TIP3P for water and modified TIP3P-HW for heavy water (95).

Each collagen triple helix was solvated in a cubic box (12 nm per edge), with TIP3P (96) or TIP3P-HW (95) water molecules, to which 140 mM NaCl was added to mimic experimental conditions. The N- and C- termini were uncapped. Periodic boundary conditions were applied and the time step was fixed to 2 fs. Following the steepest descent minimization, the system were first equilibrated under constant pressure for 5 ns, with position restraints applied on the heavy atoms of the protein, followed by 5 ns NPT equilibration in the absence of restraints. The temperature and the pressure were maintained constant at 300 K and 1 atm, respectively by using the modified Berendsen thermostat (0.1 ps coupling) (97) and Berendsen barostat (2 ps coupling) (98). The production simulations were performed in the NVT ensemble in the absence of restraints. The short-range interactions were cut-off beyond distances of 1.2 nm, and the potential smoothly decays to zero using the Verlet cut-off scheme. The Particle Mesh Ewald (PME) technique (99) was employed (cubic interpolation order, real space cut-off of 1.2 nm and grid spacing of 0.16 nm) to compute the long-range electrostatic interactions.

### Coarse Grained Molecular Dynamics Simulations.

Our model is based on the “D-mimetic” molecule, which is a synthetic collagen-mimetic molecule, that has been shown to self-assemble into collagen-like fibrils (55, 100). Since this D-mimetic protein consists of 36 amino acids only, our molecule consists of 36 beads that are arranged into a linear chain. With σ being the MD unit of length, each of these beads measures r=1.12σ in diameter and is in contact with its direct neighbors via a harmonic bond $E=\kappa_{\mathrm{bond}}{\left(r-r_{0}\right)}^{2}$, where $\kappa_{\mathrm{bond}}=500\;\mathrm{kT}/\sigma^{2}$ is the bond strength and $r_{0}=0.255\sigma$ is the equilibrium distance. This results in a molecule length of l=10σ and consequently, σ=1 nm, because the D-mimetic peptide has a length of 10 nm. We use an angular potential $E=\kappa_{\text{angle}}{\left(\theta -\theta_{0}\right)}^{2}$ that acts between three neighboring beads to define the rigidity of our molecule, where $\kappa_{\text{angle}}=50kT$ controls the molecular rigidity and $\theta_{0}=\pi$ is the equilibrium angle. Additionally, all beads carry a unit charge with respect to the charge distribution of the D-mimetic molecule, as shown in Fig. 4*A* in the main text. All the beads on different molecules are able to interact with each other via a generic, hydrophobic potential described by a cut-and-shifted Lennard-Jones potential $E_{\text{LJ}}=4\epsilon_{\text{H}}\left[{\left(\sigma /r\right)}^{12}-{\left(\sigma /r\right)}^{6}\right]+E_{\text{shift}}^{\text{LJ}}$, if two interacting beads are at a distance $r<r_{c}=2\sigma$, and is 0 otherwise, $\epsilon_{\text{H}}$ is the strength of nonspecific or hydrophobic interactions , which is one of our control parameters. Furthermore, two charged beads i,j are able to interact with each other via a cut-and-shifted screened electrostatic potential (DLVO) $E_{\text{DLVO}}=\left(\epsilon_{\text{E}}q_{i}q_{j}/r\right)\;\exp(-\kappa r)+E_{\text{shift}}^{\text{DLVO}}$, if the two beads are at a distance $r<r_{c}=2\sigma$, and 0 otherwise, κ=1σ is the screening length and its length of 1 nm corresponds to the Debye screening length at physiological conditions. $\epsilon_{\text{E}}$ defines the effective strength of the electrostatic interactions and is the second control parameter we will explore, while $q_{i}$ represents the sign of the charge of bead i ($q_{i}=\pm 1$). Since neighboring beads in a molecule have overlapping volume and distances between charges in the same molecule can be small, we exclude interactions of beads in the same molecule for 1 to 2, 1 to 3, 1 to 4, and 1 to 5 neighbors. The simulations are initialized by randomizing the positions and orientations of N=2,500 molecules in a cubic box of length L=171σ, resulting in a molecule number concentration of $c_{\text{mol}}=0.0005\sigma^{-3}$. We integrate the system at constant number of particles, N, and constant volume, V, with a Langevin thermostat to simulate Brownian motion of the molecules, with the LAMMPS MD package (101). Our integration timestep is $0.001\tau_{0}$, where $\tau_{0}$ denotes the MD unit of time, and the damping coefficient was chosen to be $1\tau_{0}$.

## Supplementary Material

Appendix 01 (PDF)

## Data Availability

Turbidity data; IR and 2D-IR data; TEM images; cryo-TEM images; CRM images; coarse grained simulations; molecular dynamics simulation data have been deposited in UvAauas.figshare (https://doi.org/10.21942/uva.24829896) (102).
